# Preterm Infant-Associated *Clostridium tertium*, *Clostridium cadaveris*, and *Clostridium paraputrificum* Strains: Genomic and Evolutionary Insights

**DOI:** 10.1093/gbe/evx210

**Published:** 2017-09-29

**Authors:** Raymond Kiu, Shabhonam Caim, Cristina Alcon-Giner, Gusztav Belteki, Paul Clarke, Derek Pickard, Gordon Dougan, Lindsay J. Hall

**Affiliations:** 1 The Gut Health and Food Safety Programme, Quadram Institute Bioscience, Norwich Research Park, Norwich, United Kingdom; 2 Norwich Medical School, Norwich Research Park, University of East Anglia, Norwich, United Kingdom; 3 Neonatal Intensive Care Unit, The Rosie Hospital, Cambridge University Hospitals NHS Foundation Trust, United Kingdom; 4 Neonatal Intensive Care Unit, Norfolk and Norwich University Hospitals NHS Foundation Trust, Norwich, United Kingdom; 5 Wellcome Trust Sanger Institute, Wellcome Genome Campus, Hinxton, United Kingdom

**Keywords:** Clostridium, functional annotation, whole genome sequencing, virulence

## Abstract

*Clostridium* species (particularly *Clostridium difficile*, *Clostridium botulinum*, *Clostridium tetani* and *Clostridium perfringens*) are associated with a range of human and animal diseases. Several other species including *Clostridium tertium*, *Clostridium cadaveris*, and *Clostridium paraputrificum* have also been linked with sporadic human infections, however there is very limited, or in some cases, no genomic information publicly available. Thus, we isolated one *C. tertium* strain, one *C. cadaveris* strain and three *C. paraputrificum* strains from preterm infants residing within neonatal intensive care units and performed Whole Genome Sequencing (WGS) using Illumina HiSeq. In this report, we announce the open availability of the draft genomes: *C. tertium* LH009, *C. cadaveris* LH052, *C. paraputrificum* LH025, *C. paraputrificum* LH058, and *C. paraputrificum* LH141. These genomes were checked for contamination in silico to ensure purity, and we confirmed species identity and phylogeny using both 16S rRNA gene sequences (from PCR and in silico) and WGS-based approaches. Average Nucleotide Identity (ANI) was used to differentiate genomes from their closest relatives to further confirm speciation boundaries. We also analysed the genomes for virulence-related factors and antimicrobial resistance genes, and detected presence of tetracycline and methicillin resistance, and potentially harmful enzymes, including multiple phospholipases and toxins. The availability of genomic data in open databases, in tandem with our initial insights into the genomic content and virulence traits of these pathogenic *Clostridium* species, should enable the scientific community to further investigate the disease-causing mechanisms of these bacteria with a view to enhancing clinical diagnosis and treatment.

## Medical Relevance

*Clostridium*, which means “a small spindle” in Greek (due to its rod-shaped morphology), is classified as a genus under the phylum Firmicutes and class Clostridia, and comprises 221 species to date (September 2017) (Parte 2014). *Clostridium* spp. are Gram-positive spore-forming anaerobes found ubiquitously in the environment (soil and water) and the intestinal tract of humans and animals (Yamagishi etal. 1964; Miwa 1975; de Vos etal. 1982). There are several significant human and animal disease causing *Clostridium* species including *Clostridium difficile* (pseudomembranous colitis), *Clostridium botulinum* (infant botulism), *Clostridium tetani* (tetanus), and *Clostridium perfringens* (acute watery diarrhea/necrotising enterocolitis [NEC]), with associated pathology ascribed to the toxins they produce (Bruggemann and Gottschalk 2004; Awad etal. 2014; Carter and Peck 2015; Sim etal. 2015). There are also several less well-studied species including *Clostridium tertium*, *Clostridium paraputrificum*, and *Clostridium cadaveris*, which have been sporadically reported in the literature to be associated with human infection.

*C. cadaveris* (formerly *Clostridium capitovale*), is thought to be a key tissue-decomposing bacterium in dead carcasses, and is generally not considered pathogenic in living individuals (Poduval etal. 1999). However, this bacterium has infrequently been associated with human systemic diseases, including intraperitoneal infection (Leung etal. 2009) and bacteremia (Poduval etal. 1999; Schade etal. 2006).

*C. tertium*, is an aerotolerant and nontoxin-producing species. During The First World War, it was the third most frequently isolated bacteria from war wounds, after *C. perfringens* and *C. sporogenes* (Henry 1917). This organism was officially recognized as a pathogen in 1963, when the first *C. tertium*-associated septicemia case was reported (King etal. 1963). *C. tertium* has also been associated with infections including peritonitis (Butler and Pitt 1982) and pneumonia (Johnson and Tenover 1988). Importantly, *C. tertium* is also linked with cattle enteritis (Silvera etal. 2003), preterm NEC (Cheah etal. 2001) and adult enterocolitis (Coleman etal. 1993).

*C. paraputrificum* has previously been isolated from formula-fed infants within their first weeks of life (Stark and Lee 1982). This pathogen has been associated with paediatric infection (sepsis) (Brook 1995), adult necrotizing enterocolitis (Shandera etal. 1988), bacteremia (Shinha and Hadi 2015), and preterm NEC (Smith etal. 2011). Interestingly, this organism was shown to produce NEC-like lesions, including gas cysts, in an animal model and thus supports their disease-causing link (Waligora-Dupriet etal. 2005).

Whole genome sequencing (WGS) has contributed significantly to biomedical and veterinary research through our increased understanding of pathogens at a genomic level. Despite the medical importance of these three pathogenic *Clostridium* species, there is currently no sequenced genomes of *C. tertium* or *C. cadaveris* available to the research community (apart from 16S rRNA gene sequences) and only four genomes of *C. paraputrificum* accessible on NCBI databases as of September 2017 (Geer etal. 2010). In this study, we sequenced one *C. cadaveris* isolate, one *C. tertium* isolate and three *C. paraputrificum* isolates from preterm infant faecal samples obtained from two neonatal intensive care units (NICUs) units in England. We identified these using their 16S rRNA gene sequences (both full-length PCR and in silico) and WGS-based k-mer phylogenetic assignment, thus contributing new genomic data on these pathogenic bacteria. We also verified their phylogenetic positions using WGS data, measured genetic distances via Average Nucleotide Identity (ANI), and performed genome-wide functional annotation (COG classification). These genomic data and analyses increases our understanding of the virulence potentials and functionalities of these pathogenic bacteria, with a future view to unraveling disease-causing mechanisms.

### Genome Description

Here, we report the release of draft genomes sequenced on Illumina HiSeq 2500 platform as stated in table 1. *C. paraputrificum* isolates have a genome size between 3.6 and 3.7 million bases and a stable GC content from 29.6 to 29.9%, which is in line with the four public genomes (Geer etal. 2010). *C. tertium* has a larger genome (3.9 million bases) and relatively lower GC content of 27.8%, whilst *C. cadaveris* has a smaller genome (3.4 million bases) compared with *C. paraputrificum*, and a significantly higher GC content of 31.2%. All draft genomes were assembled using Prokka de novo assembler and 80% (four out of five) of the genomes analyzed were <50 contigs, except for *C. paraputrificum* LH058 with 84 contigs.

**Table 1:** Genome Description, Assembly Statistics, and Clinical Information of Isolates Used in This Study

	*C. tertium*LH009	*C. cadaveris*LH052	*C. paraputrificum* LH025	*C. paraputrificum* LH058	*C. paraputrificum* LH141
Genome size (bp)	3,970,462	3,460,249	3,797,748	3,776,795	3,630,606
No. of contigs	49	46	40	84	29
Genes	3,910	3,395	3,896	3,823	3,655
CDS	3,821	3,310	3,813	3,745	3,565
N_50_ (bp)	258,765	118,391	479,233	101,241	390,404
tRNAs	89	84	83	77	90
GC content (%)	27.8	31.2	29.6	29.9	29.7
Origin of isolates	29-week preterm infant	32-week preterm infant	29-week preterm infant	32-week preterm infant	27-week preterm infant
Hospital	RH	NNUH	RH	NNUH	RH

* RH: Rosie Hospital, Cambridge, UK; NNUH: Norfolk and Norwich Hospital, Norwich, UK.

These five strains were isolated from preterm infants residing at two different NICUs (table 1), which is in line with previous findings that report frequent detection of *C. paraputrificum* (16–22%) and *C. tertium* (4–9%) in infant cohorts (Tonooka etal. 2005; Ferraris etal. 2012). However, to date there are no reports of *C. cadaveris* isolation from infants.

## Phylogenetic Positions

**Fig. 1.**
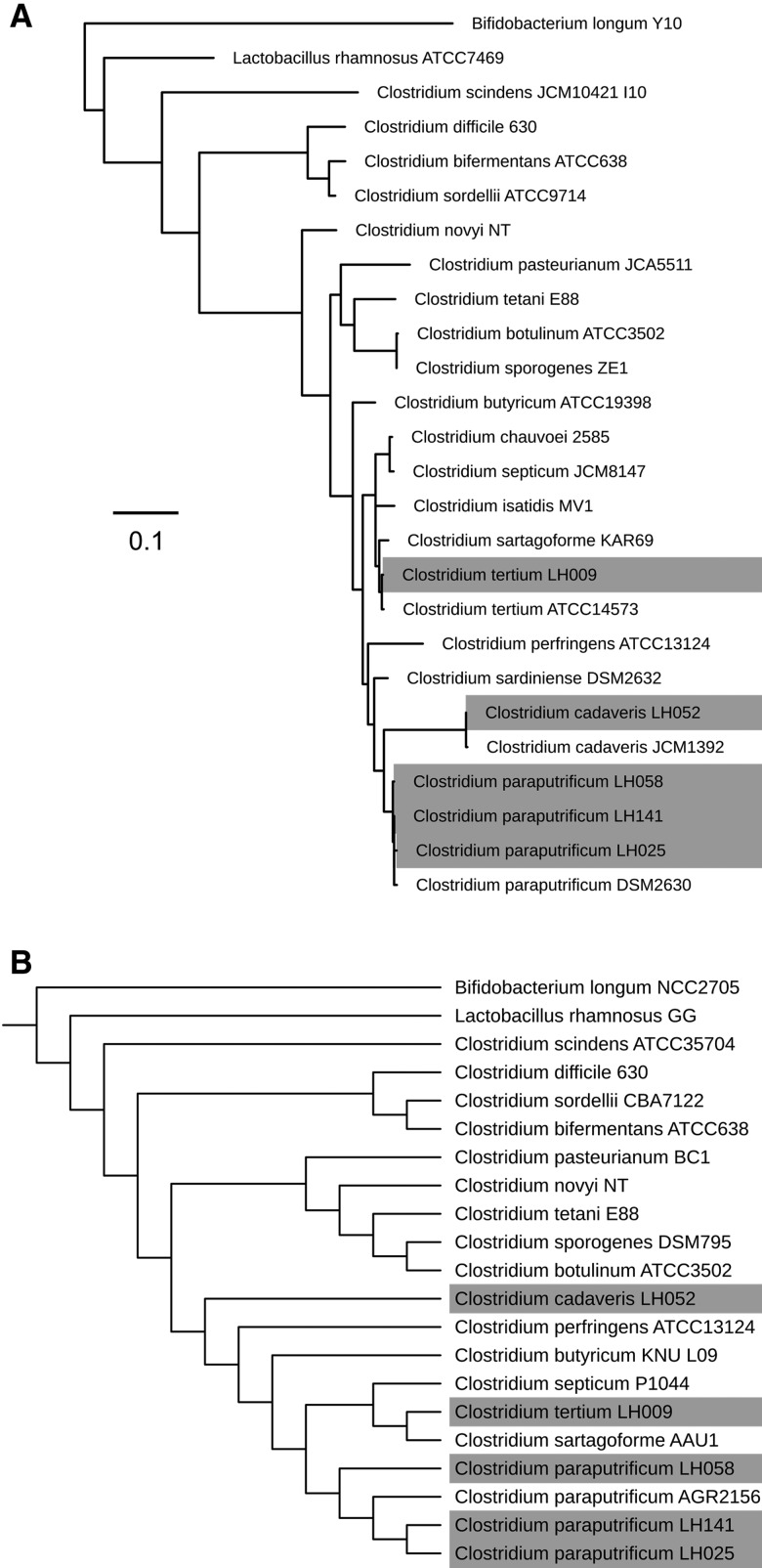
—(*A*) 16S rRNA maximum-likelihood (ML) phylogenetic tree of 19 species of *Clostridium*. (*B*) WGS-based alignment-free cladogram of representative *Clostridium* species. *Lactobacillus rhanmosus* and *Bifidobacterium longum* have been used as outgroups. Grey labels indicate newly sequenced isolates in this study.

To assign phylogenetic position, and identify these isolates, we computationally extracted 16S rRNA sequences from genomes to construct a *Clostridium* 16S rRNA phylogeny (based on 19 isolates in the NCBI nucleotide database) as in figure 1*A*. Here, we coupled three genomic approaches to confirm taxonomic position of these newly released genomes. We firstly, performed a PCR targeting almost the full length of the 16S rRNA gene, and predicted the whole 16S rRNA gene sequence in silico. Secondly, we employed Average Nucleotide Identity (ANI) to confirm species boundaries; ANI cut-offs for species discrimination is known to be approximately 95%, and this value has been reported to mirror the traditional taxonomic gold standard method DNA–DNA hybridization (DDH) to define species (Richter and Rossello 2009). Lastly, we performed CVTree—an alignment-free whole genome-based phylogenetic construction method, which is known for speed and accuracy for taxonomic assignment (Xu and Hao 2009).

At a 16S rRNA level, LH058, LH141, and LH025 fall in the same lineage as *C. paraputrificum* DSM2630, indicating species-level relatedness (fig. 1*A*), with LH052 clustering with *C. cadaveris* JCM1392, and LH009 within the same lineage as *C. tertium* ATCC14573 and *Clostridium sartagoforme* KAR69. CVTree phylogenetic analysis, providing greater resolution based on sequence comparison, showed similar relationships. (fig. 1*B*); all *C. paraputrificum* isolates grouped within the same lineage as *C. paraputrificum* DSM2630, when compared with other *Clostridium* species, indicating correct species assignment for isolates LH058, LH141, and L025. *C. cadaveris* LH052 is most closely related to *C. perfringens* ATCC13124, and LH009 (*C. tertium* as assigned according to 16S data) is closely related to *C. sartagoforme* AAU1 (fig. 1*B*).

**Fig. 2.**
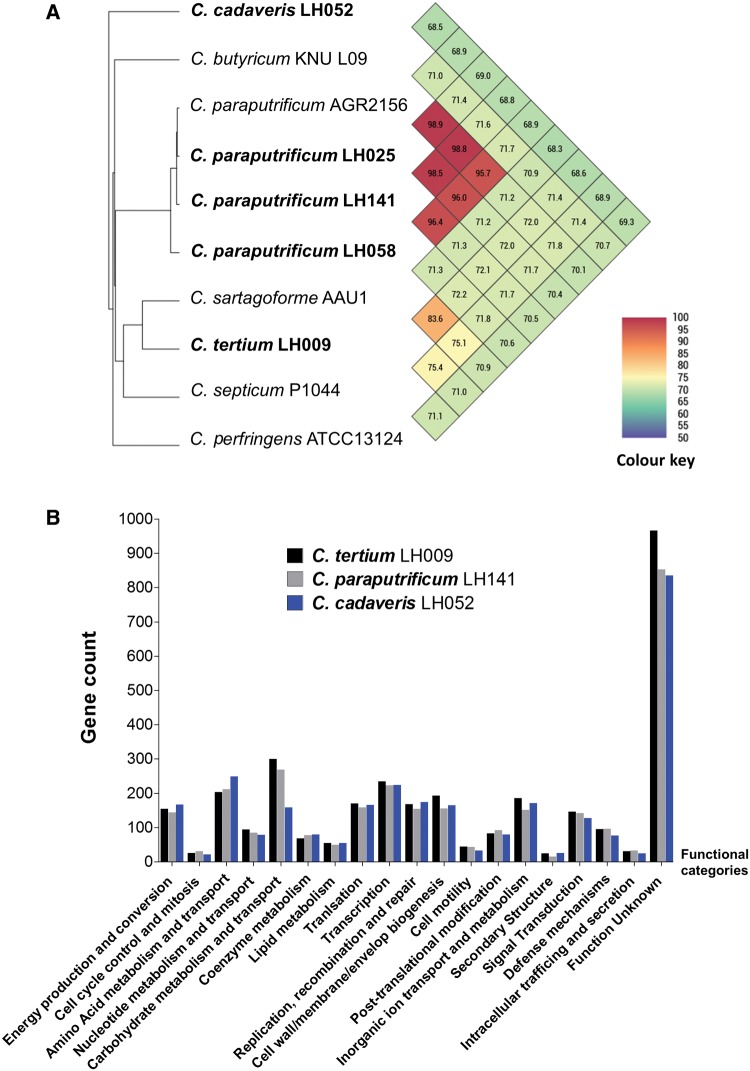
—(*A*) Average Nucleotide Identity (ANI) values in representative *Clostridium* genomes. (*B*) Comparison of functional annotations based on COG classifications on three representative genomes.

We next used ANI analysis to provide higher phylogenetic resolution (fig. 2*A*). *C. paraputrificum* AGR2156 are identical to LH025, LH141, and LH058 in terms of nucleotide sequences, sharing ANI of >95.7%, thus determined to be the same species. Although LH009 is closely related to *C. sartagoforme* AAU1, the ANI calculation does not allocate these two within the same species (ANI = 83.6%, < 95% as species cut-off), which indicates LH009 is distinct from its closest relatives, and may be identified as the species *C. tertium*. LH052 is also evolutionarily distant (based on ANI calculation, 68.5%) from other *Clostridium*, indicating this isolate is a separate species, *C. cadaveris.*

## Virulence Traits and Genome-Wide Functional Analyses

Using genome annotations, we performed a thorough search on virulence-related terms including “phospholipase,” “hemolysin,” “resistance,” “lactamase,” “drug,” and “toxin” to provide initial insights into the potential virulence-linked genes encoded within these genomes (table 2).

**Table 2:** Virulence-Related Genes Detected in Selected *Clostridium* Genomes

Isolate	Gene Names	Gene Description and Functions
*C. tertium* LH009	*ytpA*	Phospholipase
	*vanW*	Vancomycin B-type resistance
	*stp*	Multidrug resistance
	*mdtK*	Multidrug resistance
	*tetM*	Tetracycline resistance
	*marA*	Multiple antibiotic resistance
	*mecR1*	Methicillin resistance
	*norM*	Multidrug resistance
	*mepA*	Multidrug export protein
	*hcpC*	Beta-lactamase precursor
	*sme-1*	Carbapenem-hydrolyzing beta-lactamase precursor
*C. cadaveris* LH052	*ytpA*	Phospholipase
	(n/a)	Patatin-like phospholipase
	(n/a)	Phospholipase C precursor
	*toxA*	Toxin A
	*marA*	Multiple antibiotic resistance
	*vanW*	Vancomycin B-type resistance
	*norm*	Multidrug resistance protein
	*tetM*	Tetracycline resistance
	*marR*	Multiple antibiotic resistance
	*mdtA*	Multidrug resistance
	*mepA*	Multidrug export protein
	(n/a)	Beta-lactamase precursor
*C. paraputrificum* LH141	*ytpA*	Phospholipase
	*toxA*	Toxin A
	*marR*	Multiple antibiotic resistance
	*tetM*	Tetracycline resistance
	*norM*	Multidrug resistance
	*vanW*	Vancomycin B-type resistance
	*mdtK*	Multidrug resistance
	*mepA*	Multidrug export protein
	(n/a)	Beta-lactamase precursor
	*sme-1*	Carbapenem-hydrolyzing beta-lactamase precursor

*C. tertium* LH009, *C. cadaveris* LH052, and *C. paraputrificum* LH141 harbour phospholipase genes (*ytpA*) that are homologous to phospholipases encoded in other pathogen genomes including *Bacillus subtilis*, *Pseudomonas aeruginosa*, and *Streptococcus pneumoniae*. Phospholipases are known to possess hydrolytic activity against eukaryotic cell membranes, and are thus considered key virulence factors. *C. perfringens* produces homologous phospholipase C (also known as alpha toxin) that has previously been reported to damage epithelial cells (Verherstraeten etal. 2013), and which shares >58% protein sequence identity with the phospholipase encoded by gene *ytpA*. Importantly, LH052 and LH141 also possess *toxA* gene, which encodes *C. difficile-*associated Toxin A, known to be one of the main virulence factors during infection having cytotoxic and proinflammatory activities (Awad etal. 2014).

Notably, antimicrobial resistance genes are encoded in all three genomes, including vancomycin (*vanW*) and tetracycline resistance (*tetM*) (Evers and Courvalin 1996; Donhofer etal. 2012). Other resistance traits include multidrug efflux pumps; that is, those encoded by *mdtK* and *norM* (fluoroquinolones) (Horiyama etal. 2011; Golparian etal. 2014), *mdtA* (aminocoumarin) (Guerrero etal. 2012), and efflux pump transcriptional regulators *marA* and *marR* (Maira-Litran etal. 2000). In addition, methicillin resistance gene *mecR1* was detected in LH009 (Shore etal. 2011), whilst beta-lactamase (penicillins and carbapenems) precursor (inactive protein sequence that could potentially be activated via posttranslational modification) was encoded in all genomes (Marciano etal. 2007). The prevalence of multiple antimicrobial resistance genes in these clinical strains may correspond to the environment in which they were isolated; preterm infants residing in NICUs where antimicrobial usage is extensive (Albrich etal. 2004).

From the COG-based genome-wide annotation, most genes (>40% in each genome) did not map with any known functional orthologs, which highlights the limitation of genomic tools and current databases, for understanding these bacteria at a functional level. Gene counts in most categories of these three genomes did not differ significantly from one another (fig. 2*B*). However, the number of genes involved in carbohydrate metabolism and transport is lower in *C. cadaveris* LH052 (*n* = 159), than encoded in *C. tertium* LH009 (*n* = 300) and *C. paraputrificum* LH141 (*n* = 269), whereas LH052 possesses more genes (*n* = 249) involved in amino acid metabolism and transport as compared with LH009 (*n* = 203) and LH141 (*n* = 212). These functional differences may correspond to divergent modes of metabolism and nutritional substrates for *C. cadaveris*, which is distinct from *C. tertium* and *C. paraputrificum* (correlates to WGS phylogeny positions), and may link to previous isolations of this species from additional environmental niches, i.e. dead carcasses. Therefore, we conclude that these three *Clostridium* species are similar in terms of genomic functionalities, however due to the high number of function-unknown genes, this somewhat reduces in-depth comparison between genomes and will require further experimental work.

## Materials and Methods

### Faecal Sample Collection

Fecal sample collection was performed under an on-going preterm infant study (BAMBI) which is approved by University of East Anglia (UEA) Faculty of Medical and Health Sciences (FMH) Ethics Committee. Sample collection was done in accordance with the procedures outlined by National Research Ethics Service (NRES) approved UEA Biorepository (Licence no.: 11208). Participating infants were given written consent by their parents for fecal sample collection at Norfolk and Norwich University Hospital (Norwich, UK) and Rosie Hospital (Cambridge, UK). Fecal samples were routinely collected from infant nappies in the NICUs into sterile stool containers and stored at 4 °C.

### Bacterial Isolates and Preliminary 16S rRNA PCR Identification

A total of five *Clostridium* isolates (including *C. tertium*, *C. cadaveris*, and *C. paraputrificum*) were analyzed in this study. Isolates were preliminarily identified using 16S rRNA full-length PCR (Weisburg etal. 1991). Primers used as in table 3. Near 1kbp PCR products were subsequently sequenced (Eurofins, Luxembourg) and compared with 16S rRNA bacteria sequence database on NCBI using BLASTn (optimized for megablast) search algorithm (Camacho etal. 2009).

**Table 3:** Sequence of Primers Used for PCR Amplification of 16S rRNA Gene

Primers	Sequence
fD1	5′-AGA GTT TGA TCC TGG CTC AG-3′
fD2	5′-AGA GTT TGA TCA TGG CTC AG-3′
rP1	5′-ACG GTT ACC TTG TTA CGA CTT-3′

### Genomic DNA Extraction

Overnight 10 ml pure cultures in BHI were harvested for phenol-chloroform DNA extraction. Briefly, bacterial pellets were resuspended in 2 ml 25% sucrose in 10 mM Tris and 1 mM EDTA at pH 8.0. Cells were lysed using 50 μl 100 mg/ml lysozyme (Roche). 100 μl 20 mg/ml Proteinase K (Roche), 30 μl 10 mg/ml RNase A (Roche), 400 μl 0.5 M EDTA (pH 8.0) and 250 μl 10% Sarkosyl NL30 (Fisher) were added subsequently into the lysed bacterial suspension. This follows by 1-h ice incubation and 50 °C overnight water bath.

Second-day protocol comprises three rounds of phenol-chloroform-isoamyl alcohol (Sigma) extraction using 15 ml gel-lock tubes (Qiagen). Chloroform-Isoamyl alcohol (Sigma) extraction was performed to remove residual phenol, followed by ethanol precipitation and 70% ethanol wash. DNA pellets were finally resuspended in 200–300 μl of 10 mM Tris (pH 8.0). DNA concentration was quantified using Qubit dsDNA BR assay kit (Invitrogen) and DNA quality assessed by Nanodrop spectrophotometer.

### Whole Genome Sequencing, Genome Assembly and Annotation

Isolated DNA of pure cultures was subjected to multiplex standard Illumina library preparation protocol followed by sequencing via Illumina HiSeq 2500 platform with read length 2 × 125 bp (paired-end reads) and an average sequencing coverage of 60×. Draft genome assemblies were generated using an assembly and annotation pipeline as described previously (Page etal. 2016). All genomes were annotated using *Prokka* v1.11 (Seemann 2014).

### Contamination Estimation

Webtool *ContEst16S* was used to check for potential contamination of the draft genomes based on Genbank database (Lee etal. 2017).

### 16S rRNA Phylogeny

Publicly available16S rRNA genes were retrieved from NCBI nucleotide database (Geer etal. 2010). 16S rRNA genes from our isolates were predicted using *Barrnap* v0.7 (https://github.com/Victorian-Bioinformatics-Consortium/barrnap, last accessed September 20, 2017) and extracted using *BEDTools* getfasta utility (Quinlan and Hall 2010). All 16S rRNA sequences were subsequently concatenated as a multisequence fasta, and sequences were aligned with *MUSCLE* (Edgar 2004). Neighbor-joining (NJ) tree was generated in 1000 bootstrap replicates using Juke-Cantor distance (Gouy etal. 2010). Maximum-likelihood (ML) tree was produced by *PhyML* GTR model with 1000 bootstrap replicates (Guindon etal. 2010). Trees were edited using *iTOL* (Letunic and Bork 2016).

### Alignment-Free WGS Phylogeny

Selected *Clostridium* genome sequences were retrieved from NCBI genome database. Annotated multiple protein sequences were used as input for *CVTree* v5.0 to generate alignment-free WGS-based phylogeny using the optimized six as the k-tuple length (Xu and Hao 2009). Tree was edited using *iTOL* as described in previous section.

### Average Nucleotide Identity (ANI)

*OrthoANI Tool* v.093 (OAT) was employed to calculate the ANI (both directions) between genomes (Lee etal. 2016). Identity >95% was used as cut-off for species delineation.

### Genome-Wide Functional Assignment (COG)

Functional assignments were implemented using *eggNOG-mapper* v0.99.3 (Huerta-Cepas etal. 2017), based on *eggNOG* orthology data (Huerta-Cepas etal. 2016). Sequence searches were performed using HMMER3 (Eddy 2011). Data were extracted using Shell scripts (https://github.com/raymondkiu/eggnog-mapper_COGextraction, last accessed September 20, 2017) and visualized in *GraphPad PRISM* v5.04. 

### Ethics Approval and Consent for Participation

This study was approved by the University of East Anglia (UEA) Faculty of Medical and Health Sciences (FMH) Ethics Committee. Sample collection follows the protocols outlined by NRES approved UEA Biorepository (Licence no.: 11208). Written consent was given by the parents for their infants for participation in this study.
